# Flare of clonal hematopoiesis, *TP53* expansion and prior melphalan drive post-CAR-T myeloid disorders in multiple myeloma

**DOI:** 10.1038/s41375-026-02941-2

**Published:** 2026-03-24

**Authors:** Johannes Waldschmidt, David Fandrei, Judith S. Hecker, Manja Meggendorfer, Marietta Truger, Franz Reinhard, Benjamin Shibru, Marion Högner, Anna Gebauer, Max Köppel, Heiko Müller, Johannes Jung, Daniel Teschner, Max S. Topp, Anna Purcarea, Vladan Vučinić, Torsten Haferlach, Florian Bassermann, Uwe Platzbecker, Hermann Einsele, Claudia Haferlach, K. Martin Kortüm, Klaus H. Metzeler, Maximilian Merz, Katharina S. Götze, Leo Rasche

**Affiliations:** 1 Department of Internal Medicine II, University Hospital of Würzburg, Würzburg, Germany; 2 Interact Advanced Clinician Scientist Program, University Hospital of Würzburg, Würzburg, Germany; 3 Bavarian Center for Cancer Research (BZKF), Würzburg, Germany; 4 Department of Hematology, Hemostaseology, Cellular Therapy and Infectious Diseases, University Hospital Leipzig, Leipzig, Germany; 5 Department of Medicine III, Technical University of Munich, School of Medicine and Health, Munich, Germany; 6 TranslaTUM, Center of Translational Cancer Research, Technical University of Munich (TUM), Munich, Germany; 7 MLL Munich Leukemia Laboratory, Munich, Germany; 8 Department of Hematology and Medical Oncology, Georg August University Göttingen, Göttingen, Germany; 9 Mildred-Scheel-Nachwuchszentrum, University Hospital of Würzburg, Würzburg, Germany

**Keywords:** Myeloma, Immunotherapy

## To the Editor

Chimeric antigen receptor (CAR) T-cell therapies have significantly improved outcomes in relapsed/refractory multiple myeloma (RRMM). However, concerns are emerging regarding long-term toxicities, including prolonged cytopenias and an increased risk of secondary hematologic malignancies. While T-cell lymphomas have been reported to occasionally develop from insertional mutagenesis of transcriptional regulators [1, 2], the majority of secondary malignancies observed in clinical trials for MM are of myeloid origin, as shown by a 10.3% incidence of post-cytotoxic therapy myeloid neoplasms (MN-pCT) in the CARTITUDE-1 study [3]. The pathogenesis of MN-pCT is well understood and can be attributed to prior exposure to mutagenic agents such as high-dose melphalan or radiation, selective pressures including lenalidomide treatment [4], as well as to aging. However, it remains unclear if this process is additionally accelerated by the specific inflammation linked to CAR T-cell expansion within the hematopoietic niche [5]. Such dynamics may be detrimental especially in patients harboring pre-existing clonal hematopoiesis (CH) which is present in up to 21.6% of MM patients [6].

**Fig. 1:**
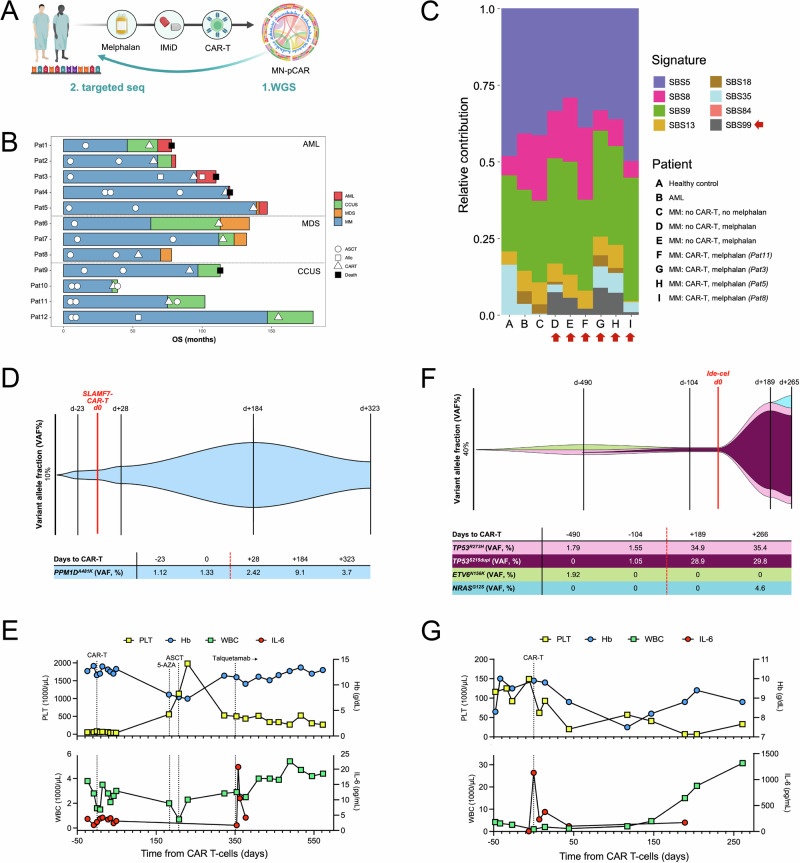
CAR T-related progression to MN-pCAR in myeloma patients with pre-disposing CH. **A**. Study design and experimental approach using WGS and panel sequencing to determine VAF expansion over time in retrospective CD138-negative BMMC samples pre- and post-CAR T-cell infusion. **B**. Swimmer plot showing disease onset and interventions. **C**. SBS99 melphalan signature (grey) in BMMCs or CD138-negative BM cell fraction including four transplant and CAR T-cell exposed MM patients with MN-pCAR from our cohort (**F**–**I**) vs. one healthy control (**A**), one AML pt (**B**), one transplant-naive MM pt (**C**) and two transplanted but CAR T-cell naïve pts (**D**, **E**). **D**. Dolphin plot and tabular clone counts for Pat11, aged 53 years, who developed *PPM1D*^A481K^-mutant CCUS at day +28 after an academic anti-SLAMF7 CAR T-cell infusion. **E**. Clinical timeline showing blood counts and inflammatory markers in Pat11. **F**. Dolphin plot for Pat1, aged 78 years, with rapid onset AML driven by *TP53*^*R273H*^ and *TP53*^*S215dup*^ mutant CH. **G**. Clinical timeline showing blood counts and inflammatory markers in Pat1. The patient died in external care at day +386 after ide-cel infusion. PLT platelets, Hb hemoglobin, WBC white blood cells (leukocytes), IL-6 interleukin-6, WGS whole-genome-sequencing, MN-pCAR myeloid neoplasm post-CAR T-cell therapy, 5-AZA 5-azacitidine, ASCT autologous stem-cell transplantation. **A** *Created in BioRender under license* https://biorender.com/2fddcbl.

In this multi-center study, we investigated inflammation-driven progression to myeloid neoplasms post-CAR T-cell therapy (MN-pCAR) as a key complication of CAR T-cell therapy. From a total of 179 patients, we identified 12 patients (6.7%, median age 58 (range 53–79) years) with myeloid clonality after CAR-T (Fig. 1A, B). All patients provided informed consent in accordance with the Declaration of Helsinki (Methods detailed in *Extended Methods*) and all analyses were approved by the local ethics board (vote 8/21) at University of Würzburg. At a median follow-up of 17.7 months post-infusion, no treatment-related lymphoid neoplasms were observed, whereas myeloid disorders included five cases of AML, three MDS and four CCUS, defined by a leukemia-associated driver mutation (variant allele frequency (VAF) ≥ 2%) and cytopenia without high-grade dysplasia (Table 1) [7]. No clear associations were observed between the type of MN-pCAR and CAR-HEMATOTOX score or inflammatory markers, possibly due to limited sample size in this study (Suppl. Table 1). Affected patients had received extensive prior treatment, including lenalidomide (12/12, 100%), a median of 2 autologous stem-cell transplants (ASCT; range 1–3) with high-dose melphalan, and allogeneic transplantation in 2 of 12 cases. In the eight patients with MDS or AML, MN-pCAR developed unexpectedly early after CAR T-cell infusion (median latency 0.4 years, range 0.1–1.3) and was observed with both commercially available BCMA-directed products (ide-cel, *n* = 5; cilta-cel, *n* = 3), preceded by standard fludarabine/ cyclophosphamide lymphodepletion.

**Table 1:** Patient characteristics (*n* = 12).

	Age at MM diagnosis (years)	Sex	Len-exposed	Pom-exposed	Thal-exposed	# Auto-SCTs	# Allo-SCTs	Type of CAR-T	Type of MN-pCAR	Alterations	Alive
Pat1	72	M	yes	yes	no	1	0	Ide-cel	AML	*TP53, NRAS, ETV6*	no
Pat2	52	M	yes	yes	no	2	0	Ide-cel	AML	*TP53, DNMT3A, PPM1D, RAD21*	yes
Pat3	49	M	yes	yes	yes	1	1 + 1*	Ide-cel	AML	*MECOM rearr*	no
Pat4	53	M	yes	yes	no	3	0	Cilta-cel	AML	*TP53, DNMT3A*	no
Pat5	62	M	yes	yes	no	2	0	Cilta-cel	AML	*TP53, DNMT3A, IDH1*	yes
Pat6	69	M	yes	yes	no	1	0	Ide-cel	MDS	*DNMT3A, TET2, MECOM rearr*	yes
Pat7	65	M	yes	yes	no	2	0	Ide-cel	MDS	*TP53, PPM1D, TET2, RUNX1, DNMT3A, BCOR*	yes
Pat8	63	M	yes	yes	no	2	0	Cilta-cel	MDS	*TP53, PPM1D, TET2, PPM1D, DNMT3A, IDH1*	yes
Pat9	58	F	yes	yes	no	2	0	Cilta-cel	CCUS	*TET2, DNMT3A*	no
Pat10	64	F	yes	yes	no	2 + 1^a^	0	Cilta-Cel	CCUS	*DNMT3A*	yes
Pat11	46	M	yes	no	no	2 + 1^a^	0	SLAMF7-CART	CCUS	*PPM1D*	yes
Pat12	59	F	yes	yes	yes	2	1	Ide-cel	CCUS	*DNMT3A*	yes

*MM* multiple myeloma, *F* female, *M* male, *Len* lenalidomide, *Pom* pomalidomide, *Thal* thalidomide, *Auto-SCT* autologous stem-cell transplant, *Allo-SCT* allogenic stem-cell transplant, *CAR-T* chimeric antigen receptor T-cell, *cilta-cel* ciltacabtagene autoleucel, *ide-cel* idecabtagene vicleucel, *MN-pCAR*= myeloid neoplasm post-CAR T-cell therapy, *AML* acute myeloid leukemia, *MDS* myelodysplastic neoplasm, *CCUS* clonal cytopenia of undetermined significance.

^a^+x in #Auto-SCT and #Allo-SCT columns indicates another ASCT or Allo-SCT conducted after CAR-T-cell therapy.

This short latency suggests that CAR T-cell therapy may directly promote myeloid acceleration, although it could also be confounded by the cumulative effects of extensive prior treatment in these late-stage patients. To address this, we analyzed a control cohort of non-CAR T-cell treated patients who developed MN-pCT after ASCT and lenalidomide maintenance (*n* = 19; Suppl. Table 2). The median latency between ASCT and myeloid malignancy in this control subgroup was 3.4 (range 0.5–12.4) years as compared to 7.5 (range 4.3–11.4) years in CAR T-cell exposed patients. Consistently, a recent Mayo Clinic study reported only modest CH expansion after ASCT, supporting the hypothesis that CAR T-cell therapy may be needed as a second stimulus in the context of intensive pre-treatment to drive expansion of preselected CH clones in MM.

In 8/12 cases of our cohort pre-CAR T-cell samples were available to investigate if MN-pCAR originated from pre-existing CH. Recurrently altered genes included *DNMT3A* (*n* = 9), *TP53* (*n* = 6) and *TET2* (*n* = 4), with additional mutations in *PPM1D* (*n* = 4), *IDH1* (*n* = 2), *RUNX1* (*n* = 1), *BCOR* (*n* = 1) and *ETV6* (*n* = 1). *TP53* alterations were strongly enriched in MDS and AML patients, with 6 out of 8 cases harboring *TP53* single nucleotide variants. In 2 patients, a second (subclonal or co-dominant) *TP53* mutation was detected. All patients carried a complex karyotype, including one case of AML with *MECOM* rearrangement as another strong driver event. A detailed synopsis of molecular characteristics is shown in Supplementary Table 3. Whole-genome-sequencing data on bone marrow mononuclear cells (BMMCs) from Pat3, Pat5, Pat8, Pat11 and five respective control individuals showed presence of SBS99 (Fig. 1C), a mutational signature previously linked to melphalan exposure [8]. This finding indicates that leukemic precursor clones were already present at ASCT and persisted beyond CAR T-cell therapy. To assess clonal dynamics in relation to CAR T-cell therapy, we tracked variant allele frequencies (VAFs) in longitudinal CD138-negative BMMC samples from 10 patients (2 not available) using informed targeted sequencing. This analysis confirmed expansion of pre-existing CH following CAR T-cell infusion. Notably, clonal trajectories followed a bimodal pattern: age-related CH mutations showed modest expansion with a transient flare post-CAR, followed by a decline upon hematopoietic recovery. For example, in Pat11 with *PPM1D*-mutated CCUS, the VAF peaked at 9.1% on day 184 after infusion of an academic anti-SLAMF7 CAR T-cell product and declined to 3.7% by day 323 post-infusion (Fig. 1D). Clonal contraction coincided with a third autologous stem-cell transplant which was conducted in this patient at day 185 post-infusion and which resulted in full stabilization of blood counts (Fig. 1E). The patient currently remains on long-term treatment with the anti-GPRC5D bispecific antibody talquetamab with ongoing complete remission for the past 2.4 years. While these findings remain anecdotal, they may support a model in which BM inflammation temporarily favors CH expansion, but as hematopoiesis recovers and inflammatory pressure subsides, the competitive advantage of clones diminishes, leading to clonal contraction. In contrast to *PPM1D*- and *DNMT3A*-mutated CH, our observations in CH involving *TP53* alterations indicated rapid CH expansion and early onset of AML. In Pat1, who tested positive for a *TP53*^*R273H*^ and *TP53*^*S215dup*^ mutation, VAFs rose from 1.55% and 1.05% at day -104–34.9% and 28.9% at day +189, respectively (Fig. 1F). Different from non-*TP53*-mutant patients, blood counts in this patient gradually deteriorated (Fig. 1G) and the patient eventually died at day +386 after ide-cel. A similar trajectory was observed in Pat2, Pat4, Pat5, Pat7, and Pat8 who also harbored *TP53*-mutant t-CH (Suppl. Table 3).

Our study aligns with a recent report by Avigan et al., which linked baseline inflammation and endothelial dysfunction to the risk of hematologic toxicity following anti-BCMA CAR T-cell therapy [9]. In a large dataset, the authors described that pre-existing *TP53*-mutated CH clones may expand from a median VAF of 3.4% pre- to 44.0% post-CAR T-cell infusion. Our findings build on this seminal work, highlighting that the origins of MN-pCAR can be traced back to melphalan treatment. This suggests that melphalan and subsequent CAR T-cell therapy are both critical to the development of MN-pCAR. Whether CH contributes to long-term cytopenia beyond established predictors such as the CAR-HEMATOTOX score still remains unclear [10], and requires further validation in prospective trials, in particular since both the study by Avigan et al. and our work did not routinely perform baseline screening for CH in unselected patients before CAR T-cell infusion and the impact of CH, in particular *TP53*, may be overestimated in both studies. In another critical analysis by Gustine et al., CH was linked to prolonged cytopenia after CAR T-cell therapy, irrespective of the CAR T-cell product used, and 2/57 CH-positive MM patients developed MDS or AML at a median follow-up of 12 months after infusion [11].

Potential mitigation strategies for clinical management may include maintenance therapy with more selective cereblon modulators, such as pomalidomide and iberdomide, which exert less selective pressure on *TP53*-mutant CH by sparing CK1α degradation [4].

Moving CAR T-cell therapy to earlier treatment lines depicts another strategy to decrease the rate of MN-pCAR. Prevalence of CH increases by ~4-fold from newly diagnosed (5.8%) to RRMM stage (25%) [12], and this is being supported by substantially lower MN-pCAR rates of only 3/208 (1.4%) cases in the CARTITUDE-4 trial with less heavily pre-treated MM patients [13]. Melphalan remains a critical driver of clonal aging of hematopoietic stem cells [14], given that melphalan-induced selection of *TP53* variants enables acquisition of additional genomic instability [15], as proposed by the high coincidence of *TP53* alterations and karyotypic abnormalities in our study. Consequently, repositioning of CAR T-cells ahead of ASCT, as currently investigated by the phase-3 CARTITUDE-6 trial for newly diagnosed MM patients, seems ultimately warranted to minimize the risk of MN-pCAR, particularly in known carriers of *TP53*-mutant CH.

## Supplementary information

Supplemental Material
