# Supplementary material for: Flare of clonal hematopoiesis, TP53 expansion and prior melphalan drive post-CAR-T myeloid disorders in multiple myeloma
Source: Leukemia. 2026 Mar 24;40(5):1054–7. doi: 10.1038/s41375-026-02941-2 (PMC13149010; doi:10.1038/s41375-026-02941-2)
Supplement: Supplementary file 1 — Supplemental Material [file 41375_2026_2941_MOESM1_ESM.docx]

**Flare of clonal hematopoiesis, *TP53* expansion and prior melphalan drive post-CAR-T myeloid disorders in multiple myeloma**

**SUPPLEMENTARY**

**R2 version**

**Authors:**

Johannes Waldschmidt^1,2,3*^, David Fandrei^4*^, Judith S. Hecker^3,5*^, Manja Meggendorfer^6^, Marietta Truger^6^, Franz Reinhard^1,3^, Benjamin Shibru^4^, Marion Högner^3,5^, Anna Gebauer^1,3^, Max Köppel^1,3^, Heiko Müller^6^, Johannes Jung^3,5^, Daniel Teschner^1,3^, Max S. Topp^1,3^, Anna Purcarea^3,5^, Vladan Vučinić^4^, Torsten Haferlach^6^, Florian Bassermann^3,5^, Uwe Platzbecker^4^, Hermann Einsele^1,3^, Claudia Haferlach^6^, K. Martin Kortüm^1,3^, Klaus Metzeler^4^, Maximilian Merz^4§^, Katharina S. Götze^3,5§^, Leo Rasche^1,3,7§^

**Affiliations:**

^1^Department of Internal Medicine II, University Hospital of Würzburg, Germany

^2^Interact Advanced Clinician Scientist Program, University Hospital of Würzburg, Germany

^3^Bavarian Center for Cancer Research (BZKF), Germany

^4^Department of Hematology, Hemostaseology, Cellular Therapy and Infectiology, University Hospital of Leipzig, Germany

^5^Department of Medicine III, Technical University of Munich, School of Medicine and Health Germany

^6^MLL Munich Leukemia Laboratory, Munich, Germany.

^7^Mildred-Scheel-Nachwuchszentrum, University Hospital of Würzburg, Germany

^*^These authors contributed as shared first authors

^§^These authors contributed as shared last authors

**Correspondence:**

Johannes Waldschmidt (waldschmid_j@ukw.de)

**Supplementary Table 1. CAR-T related safety and efficacy data (n=12)**

|  | **Baseline value** | | | | | | | | | **Post-CAR-T values** | | |
| --- | --- | --- | --- | --- | --- | --- | --- | --- | --- | --- | --- | --- |
|  | **Age at MN-pCAR diagnosis** | **EMD** | **Response prior CAR-T** | **PLT (n*1000/µl)** | **ANC (n*1000/µl)** | **Hb**  **(g/dl)** | **CRP (mg/dl)** | **Ferritin (µg/l)** | **CAR-HEMATOTOX** | **IL-6 (pg/ml)** | **CRS** | **Best response** |
| Pat1 | 78 | no | PR | 154 | 2.94 | 10.2 | 0.1 | 755 | 2 | 19,246 | 2 | PR |
| Pat2 | 58 | yes | VGPR | 81 | 1.8 | 7.9 | 9.9 | NA* | 3** | 1,915 | 2 | CR |
| Pat3 | 57 | yes | PD | 90 | 3.85 | 8.8 | 5.21 | 4,494 | 5 | 13,904 | 2 | PR |
| Pat4 | 63 | no | PR | 151 | 0.6 | 10.0 | 0.38 | 225 | 2 | 920 | 2 | PR |
| Pat 5 | 73 | yes | VGPR | NA* | NA* | NA* | NA* | NA* | NA* | NA* | NA* | CR |
| Pat6 | 79 | no | PD | 90 | 1.4 | 12.4 | 1.3 | 161 | 1 | 1,804 | 1 | CR |
| Pat7 | 75 | yes | PD | 20 | 1.31 | 7.0 | 22.9 | 922 | 5 | 94,9 | 1 | PD |
| Pat 8 | 67 | yes | PR | 280 | 3.23 | 9.5 | 0.16 | 393 | 0 | 13.7 | 0 | CR |
| Pat9 | 66 | no | VGPR | 232 | 1.43 | 8.5 | 18.6 | 228 | 2** | NA* | 0 | CR |
| Pat10 | 67 | no | PD | 17 | 5.51 | 8.4 | 5.48 | 1,254 | 5 | 3,240 | 2 | VGPR |
| Pat11 | 53 | no | PR | 122 | 1.87 | 12.7 | 0.1 | 56 | 1 | 12.6 | 0 | PD |
| Pat12 | 72 | yes | PR | 189 | 4.1 | 8.6 | 5.06 | 568 | 2 | 10,322 | 2 | CR |

# Abbreviations: MN-pCAR= myeloid neoplasm post-CAR T-cell therapy, EMD= extramedullary disease, CAR-T= chimeric antigen receptor T cells, PLT= platelet count, ANC= absolute neutrophil count, Hb= hemoglobin, CRP= C-reactive protein, IL-6= interleukin-6, CRS= cytokine release syndrome, PD/ SD/ PR/ VGPR/ CR= progressive disease/ stable disease/ partial response/ very good partial response/ complete response according to International Myeloma Working Group (IMWG) Response Criteria, **NA= not assessed/ reported, external CAR-T infusion, **CAR-HEMATOTOX score calculated with censored values*

**Suppl. Table 2. MN-pCT (n=41) outcomes in transplanted and CAR-T exposed MM patients**

|  | **Non-transplanted/ non-CAR-T exposed (n=14)** | | **Transplanted/ non-CAR-T exposed (n=19)** | | **Transplanted/ CAR-T exposed (n=8)** | |  |
| --- | --- | --- | --- | --- | --- | --- | --- |
| Age at MM (median, range [yrs]) | | 71.4 (59.5-78.0) | | 61.3 (38.2-76.2) | | 62.6 (49.6-72.7) | |
| Number of ASCTs (mean, range) | | NA | | 1.9 (1-3) | | 1.8 (1-3) | |
| Exposure to IMiDs | |  | |  | |  | |
| lenalidomide | | 3 (21%) | | 17 (89%) | | 8 (100%) | |
| pomalidomide | | 0 (0%) | | 4 (21%) | | 8 (100%) | |
| thalidomide | | 0 (0%) | | 4 (21%) | | 1 (13%) | |
| Age at MN (median, range [yrs]) | | 75.1 (63.4-81.6) | | 66.6 (52.7-80.1) | | 71.3 (57.7-79.7) | |
| Subtype | |  | |  | |  | |
| AML | | 2 (14%) | | 8 (42%) | | 5 (63%) | |
| MDS | | 12 (86%) | | 11 (58%) | | 3 (38%) | |
| Latency to SPM (median, range [yrs]) | | 0.6 (0.0-17.0) | | 5.3 (1.2-19.9) | | 8.9 (5.7-11.8) | |
| Latency ASCT to SPM (median, range [yrs]) | | NA | | 3.4 (0.5-12.4) | | 7.5 (4.3-11.4) | |
| Latency CAR-T to SPM (median, range [yrs]) | | NA | | NA | | 0.4 (0.1-1.3) | |
| Median OS from MM diagnosis (yrs) | | 10.4 | | 10.0 | | NR | |
| Median OS from SPM diagnosis (yrs) | | 1.8 | | 1.4 | | 1.2 | |
| Median OS from CAR-T (median, range [yrs]) | | NA | | NA | | NR (0.2-NR) | |

# Abbreviations: MM= multiple myeloma, yrs= years, mos= months, ASCT= autologous stem cell transplantation, IMiDs= immunomodulatory agents, MN= myeloid neoplasm, ALL= acute lymphoblastic leukemia, AML= acute myeloid leukemia, MDS= myelodysplastic neoplasm, CCUS= clonal cytopenia of undermined significance, CAR-T = chimeric antigen receptor T cell, OS= overall survival, NA= not applicable

**Supplementary Table 3. Synopsis of CH expansion over time**

|  |  |  | |  | | |  |  |  |
| --- | --- | --- | --- | --- | --- | --- | --- | --- | --- |
| **Patient 1 (complex karyotype, XY)** | | | | | | | |  |  |
| ***Day +/- CAR-T*** | ***-490*** | | ***-104*** | | ***189*** | ***265*** | |  | |
| ***TP53*** *(c.818G>A; p.Arg273His)* | 1,79 | | 1,55 | | 34,9 | 35,4 | |  | |
| ***TP53*** *(c.643_645dup; p.Ser215dup)* | 0 | | 1,05 | | 28,9 | 29,8 | |  | |
| ***ETV6*** *(c.467dup; p.Asn156Lysfs*13)* | 1,92 | | 0 | | 0 | 0 | |  | |
| ***NRAS*** *(c.34G>A; p.Gly12Ser)* | 0 | | 0 | | 0 | 4,55 | |  | |

| **Patient 2 (complex karyotype, with del5q, XY)** | | | | | | | | | | |  |  |
| --- | --- | --- | --- | --- | --- | --- | --- | --- | --- | --- | --- | --- |
| ***Day +/- CAR-T*** | | | | ***68*** | | | ***341*** | | | |  |  |
| ***PPM1D*** *(c.1340del; p.Glu447Glyfs*4)* | | | | 5 | | | 0 | | | |  |  |
| ***RAD21*** *(c.1705-1G>T)* | | | | 8 | | | 0 | | | |  |  |
| ***TP53*** *(c.808T>G; p.Phe270Val)* | | | | 0 | | | 31 | | | |  |  |
| ***DNMT3A*** *(c.2729C>T; p. Ala910Val)* | | | | 0 | | | 16 | | | |  |  |
|  |  | |  | |  | | | |  |  | |  |
| **Patient 3 (*MECOM* rearr, monosomy 7, XY)** | |  | | | |  | |  | |  | | |
| ***Day +/- CAR-T*** |  |  | | | |  | |  | |  | | |
| no CH detected | NA |  | | | |  | |  | |  | | |

| **Patient 4 (complex karyotype, with del5q, XY)** | | |  |
| --- | --- | --- | --- |
| ***Day +/- CAR-T*** | ***36*** | |  |
| ***TP53*** *(c.102dup; p.Pro36Alafs*7)* | 41 | |  |
| ***TP53*** *(c.646G>A; p.Val216Met)* | 38 | |  |
| ***DNMT3A*** *(c.2726T>G; p.Phe909Cys)* | 46 | |  |
|  |  | |  |
| **Patient 5 (complex karyotype, del5q, del7q, XY)** | | | |
| ***Day +/- CAR-T*** | ***126*** | ***259*** | |
| ***DNMT3A*** *(c2401A>G; p.Met801Val)* | 47 | 39 | |
| ***IDH1*** *(c.394C>T; p.Arg132Cys)* | 7 | 13 | |
| ***TP53*** *(c814G>C, p.Val272Leu)* | 47 | 38 | |

| **Patient 6 (complex karyotype, t(3;11) not leading to *MECOM* rear, XY)** | | | | | | | | | | | | | | |  |  |  |  |  |  |  |
| --- | --- | --- | --- | --- | --- | --- | --- | --- | --- | --- | --- | --- | --- | --- | --- | --- | --- | --- | --- | --- | --- |
| ***Day +/- CAR-T*** | ***-1505*** | | ***28*** | | | | **94** | | ***595*** | | ***651*** | | | |  |  |  |  |  |  |  |
| ***DNMT3A*** *(c2141C>G; p. Ser714Cys)* | 16 | | 28 | | | | 36 | | 24 | | 19 | | | |  |  |  |  |  |  |  |
| ***TET2*** *(c.5162_5163de linsGA; p.Leu1721)* | 4 | | 2 | | | | 3 | | 1 | | 3 | | | |  |  |  |  |  |  |  |
|  |  | | | |  | | |  | | | |  |  | | | | |  |  |  |  |
| **Patient 7 (complex karyotype, XY)** | | | | | | | | | | | | |  | | | | |  |  |  |  |
| ***Day +/- CAR-T*** | ***-97*** | | | ***48*** | | | | **99** | | ***251*** | | |  |  |  |  |  |  |  |  |  |
| ***TP53*** *(c.814G>T; p.Val272Leu)* | 21,84 | | | 18,34 | | | | 30,53 | | 44,6 | | |  |  |  |  |  |  |  |  |  |
| ***TET2*** *(c.4160A>G; p.Asn1387Ser)* | 23,94 | | | 21,96 | | | | 33,58 | | 47,1 | | |  |  |  |  |  |  |  |  |  |
| ***RUNX1*** *(c.964_965delTC; p.Ser322fs)* | 20,16 | | | 14,95 | | | | 25,06 | | 44 | | |  |  |  |  |  |  |  |  |  |
| ***PPM1D*** *(c.1573G>T; p.Glu525*)* | 1,25 | | | 2,16 | | | | 1,56 | | 0 | | |  |  |  |  |  |  |  |  |  |
| ***DNMT3A*** *(c.2552delT; p.Phe851fs)* | 0 | | | 1,15 | | | | 0 | | 0 | | |  |  |  |  |  |  |  |  |  |
| ***RUNX1*** *(c.555A>C; p.Gln185His)* | 0 | | | 2,78 | | | | 2,03 | | 0 | | |  |  |  |  |  |  |  |  |  |
| ***BCOR*** *(c.4405C>T; p.Arg1469Trp)* | 0 | | | 1,01 | | | | 1,5 | | 0 | | |  |  |  |  |  |  |  |  |  |
|  |  | | | |  | | | |  | | |  |  | | | | |  |  |  |  |
| **Patient 8 (complex karyotype, XY)** | | | | | | | | |  | | |  |  | | | | |  |  |  |  |
| ***Day +/- CAR-T*** | | ***596*** | | | | ***632*** | | |  | | | | | |  | |  | |  |  |  |
| ***DNMT3A*** *(c.2173G>T; p.Glu725*)* | | 7 | | | | 9 | | |  | | | | | | |  | | | |  | |
| ***IDH1*** *(c237T>G; pAsp79Glu))* | | 0 | | | | 17 | | |  | | | |  | | | |  | | | |  |
| ***MPL*** *(c.1772T>A; p.Tyr591Asn)* | | 5 | | | | 8 | | |  | | | |  | | | |  | | | |  |
| ***PPM1D*** *(c.1538del; p.Leu513*)* | | 2 | | | | 3 | | |  | | | |  | | | |  | | | |  |
| ***TET2*** *(c.252del; pTyr85Ilefs*10)* | | 0 | | | | 4 | | |  | | | |  | | | |  | | | |  |
| ***TP53*** *(c.375G>A, p.splice site mutation)* | | 0 | | | | 5 | | |  | | | |  | | | |  | | | |  |

| **Patient 9 (normal karyotype, XX)** | | | | | | |  | |  |  | | | |  |  |  |  |  |
| --- | --- | --- | --- | --- | --- | --- | --- | --- | --- | --- | --- | --- | --- | --- | --- | --- | --- | --- |
| ***Day +/- CAR-T*** | | ***-77*** | | | ***183*** | |  | | | |  | |  | | |  |  |  |
| ***DNMT3A*** *(c.1280_1281insGAGGCACCTGCTTTCCTCCTCCAGA)* | | 1,24 | | | 0 | |  | | | | |  | | | | |  | |
| ***TET2*** *(c.3256delA; p.Thr1086fs)* | | 0 | | | 2,8 | |  | | |  | | |  | | | | |  |
|  |  | | |  | | |  |  | |  | | | | |  |  |  |  |
| **Patient 10 (normal karyotype, XX)** | | | | | | | |  | |  | | | | |  |  |  |  |
| ***Day +/- CAR-T*** | ***-26*** | | ***-5*** | | | **390** | |  | |  | | | | |  |  |  |  |
| ***DNMT3A*** *(c.1598A>G; p.Tyr533Cys)* | 3,99 | | 1,98 | | | 2,0 | |  | |  | | | | |  |  |  |  |

| **Patient 11 (normal karyotype, XY)** | | | | | | | | | |
| --- | --- | --- | --- | --- | --- | --- | --- | --- | --- |
| ***Day +/- CAR-T*** | ***-23*** | ***-8*** | | **28** | | **184** | | **323** | |
| ***PPM1D*** *(c.1439_1440dup; p.Ala481Lysfs*3)* | 1,12 | 1,33 | | 2,42 | | 9,1 | | 3,7 | |
|  |  |  | |  | |  | |  | |
| **Patient 12 (normal karyotype after allo-SCT, XY)** | | | | |  | |  | |  |
| ***Day +/- CAR-T*** | ***-259*** | | ***735*** | |  | |  | |  |
| ***DNMT3A*** *(c.2645G>T; p.Arg882Leu)* | 4,6 | | 13 | |  | |  | |  |
|  |  | |  | |  | |  | |  |

**Supplementary Table 4. Target regions of custom myeloid gene panel**

| Gene Symbol | Ensembl ID | Exons / Region of Interest |
| --- | --- | --- |
| *ASXL1* | ENSG00000171456 | E12, E13 |
| *ASXL2* | ENSG00000143970 | E12, E13 |
| *ATRX* | ENSG00000085224 | E01–E35 |
| *BCOR* | ENSG00000183337 | E02–E15 |
| *BCORL1* | ENSG00000085185 | E01–E12 |
| *BRAF* | ENSG00000157764 | E11, E15 |
| *CALR* | ENSG00000179218 | E09 |
| *CBL* | ENSG00000110395 | E01–E16 |
| *CEBPA* | ENSG00000245848 | E01 |
| *CSF3R* | ENSG00000119535 | E14–E17 |
| *CSNK1A1* | ENSG00000113712 | E03–E04 |
| *CUX1* | ENSG00000257923 | E01–E24 |
| *DDX41* | ENSG00000183258 | E01–E17 |
| *DNMT3A* | ENSG00000119772 | E02–E23 |
| *ETNK1* | ENSG00000139163 | E03–E04 |
| *ETV6* | ENSG00000139083 | E01–E08 |
| *EZH2* | ENSG00000106462 | E02–E20 |
| *FBXW7* | ENSG00000109670 | E02–E12 |
| *FLT3* | ENSG00000122025 | E14–E20 |
| *GATA1* | ENSG00000102145 | E02–E06 |
| *GATA2* | ENSG00000179348 | E02–E06 |
| *GNB1* | ENSG00000078369 | E03–E11 |
| *IDH1* | ENSG00000138413 | E04, E07 |
| *IDH2* | ENSG00000182054 | E04, E07 |
| *IL6R* | ENSG00000160712 | rs2228145 |
| *JAK2* | ENSG00000096968 | E03–E25 |
| *KIT* | ENSG00000157404 | E01–E21 |
| *KRAS* | ENSG00000133703 | E02–E05 |
| *MPL* | ENSG00000117400 | E01–E12 |
| *MYD88* | ENSG00000172936 | E01–E06 |
| *NF1* | ENSG00000196712 | E01–E58 |
| *NOTCH1* | ENSG00000148400 | E26–28, E34 |
| *NPM1* | ENSG00000181163 | E11 |
| *NRAS* | ENSG00000213281 | E02–E05 |
| *PDGFRA* | ENSG00000134853 | E02–E23 |
| *PDGFRB* | ENSG00000113721 | E02–E23 |
| *PHF6* | ENSG00000156531 | E02–E10 |
| *PIGA* | ENSG00000165195 | E02–E06 |
| *PPM1D* | ENSG00000170836 | E01–E06 |
| *PRPF8* | ENSG00000174231 | E01–E42 |
| *PTEN* | ENSG00000171862 | E01–E09 |
| *PTPN11* | ENSG00000179295 | E01–E15 |
| *RAD21* | ENSG00000164754 | E02–E14 |
| *RUNX1* | ENSG00000159216 | E01–E06 |
| *SETBP1* | ENSG00000152217 | E04 |
| *SF1* | ENSG00000168066 | E01–E13 |
| *SF3A1* | ENSG00000099995 | E01–E16 |
| *SF3B1* | ENSG00000115524 | E13–E16 |
| *SH2B3* | ENSG00000111252 | E02–E08 |
| *SMC1A* | ENSG00000072501 | E01–E25 |
| *SMC3* | ENSG00000108055 | E01–E29 |
| *SRSF2* | ENSG00000161547 | E01 |
| *STAG2* | ENSG00000101972 | E03–E35 |
| *SUZ12* | ENSG00000178691 | E01–E16 |
| *TET2* | ENSG00000168769 | E03–E11 |
| *TP53* | ENSG00000141510 | E02–E11 |
| *U2AF1* | ENSG00000160201 | E02, E06 |
| *U2AF2* | ENSG00000063244 | E02, E06 |
| *UBA1* | ENSG00000130985 | E02–E26 |
| *WT1* | ENSG00000184937 | E07, E09 |
| *ZEB2* | ENSG00000169554 | E02–E10 |
| *ZRSR2* | ENSG00000169249 | E01–E11 |

**Extended Methods**

**Whole genome sequencing (WGS)**

Whole genome sequencing (WGS) libraries were prepared from 1µg of DNA with the TruSeq PCR free library prep kit following the manufacturer’s recommendations (Illumina, San Diego, CA, USA) and 2x150bp paired-end sequences were generated on a NovaSeq 6000 instrument with 100x coverage (Illumina, San Diego, CA, USA). Reads were aligned to the human reference genome (GRCh37, Ensembl annotation) using the Isaac aligner (v3.16.02.19) [1] through BaseSpace’s WGS app (v5, Illumina, San Diego, CA, USA) with default parameters. Resulting BAM files were used in BaseSpace’s Tumor/Normal app (v3) to call single nucleotide variants (SNV) and small indels (<50bp) with Strelka (v2.4.7) [2] and large scale structural variants (SV) with Manta (v0.28.0) [3]. As no sample specific normal tissue was available, a so-called unmatched normal was used in its place to reduce technical artefacts and germline calls. For this WGS was performed on gender-matched genomic DNA from a mixture of multiple anonymous donors (Promega, Fitchburg, WI, USA). To further remove potential germline variants, each SNV/small indel was queried against the gnomAD database (v2.1.1) [4] and variants with global population frequencies >0.05% where excluded. Further analysis was performed on protein-altering and splice-site variants only. Copy number variations (CNV) were called with GATK (v4.0.8.1) [5] using the Broad Institute’s recommended best practices pipeline. Here two panels of normals (PON) were used for denoising consisting of 124 female and 191 male samples which presented a normal karyotype during routine diagnostics. Copy neutral loss of heterozygosity (CN-LOH) was assessed using HadoopCNV [6].

[1] https://doi.org/10.1093/bioinformatics/btt314

[2] https://doi.org/10.1038/s41592-018-0051-x

[3] https://doi.org/10.1093/bioinformatics/btv710

[4] https://doi.org/10.1038/s41586-020-2308-7

[5] Van der Auwera GA & O'Connor BD. (2020). Genomics in the Cloud: Using Docker, GATK, and WDL in Terra (1st Edition). O'Reilly Media. (https://www.oreilly.com/library/view/genomics-in-the/9781491975183/)

[6] https://doi.org/10.1101/124339

**Targeted myeloid panel sequencing**

The library preparation for enrichment was performed with 100ng DNA per sample with the Illumina DNA Prep Kit (Illumina, San Diego, CA, USA) using Unique Dual Indices (UDI). Within the protocol, the DNA was fragmented enzymatically to a length of 180bp. Subsequently, the DNA target regions were enriched using the IDT Hybridization Capture Protocol and a corresponding lockdown gene panel (IDT Integrated DNA Technologies, Coralville, IA, USA). Sequencing of the libraries was performed on Illumina NovaSeq X plus instruments (Illumina, San Diego, CA, USA) with paired end sequencing mode (2x101 cycles) and a target coverage of 1,500x. The custom designed lockdown panel covered the genes/regions of interest given in Supplementary Table 4. Ilumina’s BaseSpace Enrichment app (v3.1.1) was used to align the raw reads to hg19 reference sequence (Isaac Aligner v03.16.02.20). Subsequently, variants were called using PISCES (v5.1.3.60) somatic variant caller with 2% variant allele frequency (VAF) cutoff and 29 base quality filter and PCR duplicate flagging. The classification of the variants in mutated (tier 1 and 2), variant of uncertain significance (VUS), or polymorphism was done using the public databases ClinVar, COSMIC, dbSNP, gnomAD, as well as the MLL in-house variant data base.

**Targeted amplicon sequencing**

Target sequencing regions were amplified and barcoded for sequencing using the FastStart High Fidelity System or GC-RICH PCR-System (Roche Diagnostics GmbH, Mannheim, Germany). Sequencing was performed on the Illumina MiSeq (San Diego, CA, USA) with a minimum coverage of 800x and analyzed with the JSI module SeqNext (version 4.4.0; JSI medical systems GmbH, Kippenheim, Germany).

**Data analysis**

Data were analysed and figures were generated using R version 4.5 and the *swimplot* (version 1.2), *ggplot2* (version 4.0), *clevRvis* (version 1.8) and *forcats* (version 1.0) package from R.
